# Process evaluation of school-based peer education for HIV prevention among Yemeni adolescents

**DOI:** 10.1080/17290376.2012.745294

**Published:** 2013-06-18

**Authors:** Buthaina Al-Iryani, Huda Basaleem, Khaled Al-Sakkaf, Gerjo Kok, Bart van den Borne

**Keywords:** school-based intervention, peer education, HIV prevention, process evaluation, Diffusion of Innovation, Yemen, programme au sein de l'école, l'éducation donnée par les pairs, la prévention du VIH, l'évaluation du processus éducatif, la diffusion des innovations, le Yémen

## Abstract

In 2005, a survey was conducted among all the 27 high schools of Aden, which revealed low levels of knowledge on major prevention measures, and a high level of stigma and discrimination towards people living with HIV (PLWH). The results served as a baseline for implementing a school-based peer education intervention for HIV prevention in the 27 schools of Aden. In 2008, and after 3 years of implementation, a quasi-experimental evaluation was conducted, which revealed that the peer education intervention has succeeded in improving HIV knowledge and skills; and in decreasing stigmatization of PLWH. This process evaluation aims to give a deeper understanding of the quasi-experimental evaluation which was conducted in the 27 high schools of Aden, and to highlight the factors that facilitated or inhibited school peer education in such a conservative Muslim setting. Qualitative methodologies were pursued, where 12 focus group discussions and 12 in-depth interviews were conducted with peer educators, targeted students, school principals, social workers, and parents of peer educators. Results revealed that school-peer education was well received. There was an apparent positive effect on the life skills of peer educators, but the intervention had a lesser effect on targeted students. Key enabling factors have been the high quality of training for peer educators, supportive school principals, and acceptance of the intervention by parents. These findings are important for improving the life skills and peer education intervention at the school level, and in better planning and implementation of life skills and peer programmes at a national scale.

## Abstract (FR)

En 2005, une enquête a été réalisée parmi les 27 écoles secondaires d'Aden. Elle a révélé un faible niveau des connaissances concernant les principales mesures de prévention et un niveau élevé de stigmatisation et de discrimination envers les personnes vivant avec le VIH. Les résultats ont servi de base pour le développement d'un programme d'éducation par les pairs portant sur la prévention du VIH dans les 27 écoles secondaires d'Aden. En 2008, et 3 ans après l'implantation, une évaluation quasi expérimentale a été réalisée. Les résultats indiquent que le programme d'éducation a amélioré les connaissances et les compétences relatives au VIH et a diminué la stigmatisation envers les personnes vivant avec le VIH. Cette évaluation a pour objectif d'approfondir la compréhension de l'évaluation quasi expérimentale, effectuée dans les 27 écoles secondaires d'Aden et d'identifier les éléments qui ont facilité ou entravé l'éducation par les pairs au sein de l'école, dans un contexte musulmane conservateur. Des méthodologies qualitatives ont été utilisées, sous forme de 12 discussions de groupe et 12 entretiens individuels avec des éducateurs de pairs, des étudiants du groupe ciblé, des directeurs d'école, des travailleurs sociaux et des parents des éducateurs de pairs. Les résultats ont montré que l'éducation par les pairs au sein de l'école a été bien accueillie. Il y avait un effet positif sur les capacités relationnelles des éducateurs de pairs, mais l'intervention avait un effet moins important sur les étudiants ciblés. Les facteurs-clés de réussite ont été la formation de haute qualité des éducateurs de pairs, le soutien des directeurs d'école et l'acceptation du programme d'éducation par les parents. Ces résultats sont importants pour améliorer les capacités relationnelles et le programme d'éducation par les pairs au sein de l'école ainsi que pour planifier l'implantation de ce type de programme à l'échelle nationale.

## Introduction

The Republic of Yemen is located in the southern part of the Arabian Peninsula and is surrounded by Oman to the east, Saudi Arabia to the north, the Red Sea to the west, and the Arabian Sea and the Gulf of Aden to the south. Yemen is the poorest country in the Middle East and one of the poorest countries in the world (Assaad, Barsoum, Cupito & Egel 2009), where more than 45% of the population live in poverty (UNDP 2010). Yemen's population is around 22.5 million people, of which 67.3% is younger than 25 years (Central Statistics Organization 2009). Limited employment opportunities have forced many Yemenis to migrate for work to neighbouring countries, leaving their families behind (Al-Iryani, Basaleem, Al-Sakkaf, Crutzen, Kok & van den Borne 2011). Because of its proximity to conflict-affected countries in the Horn of Africa, Yemen is hosting hundreds of thousands of refugees (Al-Iryani *et al.* 2011). Yemen also faces one of the largest gender gaps in human development in the world (Assaad *et al.* 2009) and continues to occupy the last place in the gender gap index rankings of 134 countries and remains the only country in the world to have closed less than 50% of its gender gap (Hausmann, Tyson & Zahidi 2009).

Epidemiologically, the HIV prevalence among the general population of Yemen, including youth is 0.2% (UNAIDS 2010). However, high rates of poverty, unemployment, mobility, and gender disparities could make the predominantly young Yemeni population vulnerable to HIV infection (Al-Iryani *et al.* 2011). In addition, Yemeni youth, just like other youth in the Middle East and North Africa, are experiencing increased premarital sex, peer pressure to engage in risky behaviour, and changing lifestyle norms (Abu-Raddad, Akala, *et al.* 2010; Abu-Raddad, Hilmi, *et al.* 2010).

Aden, a governorate in the Republic of Yemen, located on the Arabian Sea, with close proximity to the Horn of Africa, is especially at risk of HIV infection. Previous studies have revealed that several risk factors exist in Aden, which include the presence of commercial sex work, male-to-male sex, and population movements to and from countries with high HIV prevalence (Al-Serouri, Anaam, Al-Iryani, Ramaroson & Al-Deram 2010; Busulwa, Takiyaddin, Azzubeidi, El Zein El Mousaad, Tawillah & Ziady 2006).

In March-May 2005, a survey among Yemeni high school students in Aden revealed that only 49.4% recognized the protective role of condoms (Al-Iryani, Raja'a, Kok & van den Borne 2009–2010). Stigma and discrimination levels were high among students. Around 40% of the suggested actions by students on how to deal with people living with HIV (PLWH) were punishment actions, which included suggestions for killing PLWH. The results from this study convinced the education authorities in Aden, Yemen, to implement a school-based peer education programme for HIV prevention, which focused on decreasing the level of stigma and discrimination towards PLWH and on increasing the knowledge about modes of HIV transmission and prevention (Al-Iryani *et al.* 2011). Peer education was selected as the methodology for implementation as it is considered a non-traditional health education method (Merakou 2006), and because the results of the 2005 baseline revealed that schools and friends were the main source of HIV information for students (Al-Iryani *et al.* 2009–2010). The school-based peer education intervention for HIV prevention was implemented for 3 years during the period of 2005–2008.

In 2008, the evaluation of the school-based peer education intervention was conducted (Al-Iryani *et al.* 2011). Data were collected using a self-administered questionnaire from a sample of 2510 students randomly selected from the 27 high schools, which were the sites of the peer education interventions. The results revealed that the school-based peer education interventions had succeeded in improving the levels of knowledge of HIV transmission and prevention, and in decreasing the levels of misconceptions, and of stigma and discrimination towards PLWH. The evaluation demonstrated that HIV education among school adolescents is possible in very conservative settings, given the interventions are addressed in a culturally sensitive manner, and that all key stakeholders are involved in the early stages of the interventions (Al-Iryani *et al.* 2011).

This article describes the process evaluation of the school-based intervention, which was conducted after the completion of the 2008 school-based evaluation. Given the very conservative school and cultural setting of Yemen, it was important to know why the school-peer education intervention succeeded in such a difficult setting. There is limited research on process evaluation of school-based life skills and peer education programmes for HIV prevention in culturally conservative settings. Frequently, the focus has been on studying the impact of interventions on outcomes such as self-reported knowledge, attitudes, and behaviour. Much less research was conducted to document, assess, and explain how programmes were implemented.

### Objectives

The main objectives of this process evaluation are to provide a deeper understanding of the 2008 quasi-experimental study outcomes; assess acceptability and reaction among the students, school principals, social workers, and parents; and understand the enabling and constraining factors for implementation. The evaluation was mainly based on qualitative data; however, some quantitative data were used, which were previously collected during the quasi-experimental outcome study (Al-Iryani *et al.* 2011).

### The school-based intervention

#### Overview

All 27 high schools in Aden, Yemen (12 boys' schools, 14 girls' schools, and one mixed school), representing all existing high schools were the site of the intervention. The intervention included training of school coordinators, selection and training of peer educators, training of school management teams, and implementation at school level (Al-Iryani *et al.* 2011).

#### Training of school coordinators

A team of school coordinators were selected by the education office in Aden, consisting of the director of school supervision, director of school health, director of school social services, a teacher representing the school curriculum supervisory committee, and an expert on training of trainers' methodologies. The team was trained in a 9-day workshop (8 h/day) conducted by two international Arabic speaking experts in the field of training of trainers on youth reproductive health, HIV prevention, life skills, and peer education, and the training package was based on a Jordanian life skills and peer education package supported by UNICEF Middle East and North Africa Regional Office (UNICEF 2006). The training package included topics on peer education and training of trainers' methodologies, HIV, reproductive health, sexually transmitted infections, puberty and changes during adolescence, and life skills education. The team of trained school coordinators had two functions: coordinating with the schools’ managements and parents; and conducting training workshops (Al-Iryani *et al.* 2011).

#### Selection and training of peer educators

Peer educators from all 27 high schools were recruited on a voluntary basis. Among those students who volunteered, a few were selected who met the standard criteria of having good communication skills, being accepted by classmates, and had good academic achievement. The selection was done by a committee consisting of the school coordinators and school social workers. The school coordinators visited parents of selected peer educators to explain the programme and to obtain a signed consent form allowing their daughters/sons to participate. Several visits were required, especially for parents of female peer educators, as families were reluctant to have their daughters get involved in extra-curricular activities, and especially as these related to HIV and AIDS prevention. The school coordinators team had a pivotal role in advocating with families. In case of refusal by parents, another peer educator was selected. Parents' refusals only occurred in the first year (2005); from the 30 peer educators selected, three parents refused to allow their children to participate, leaving 27 students who were trained as peer educators. In the following years, there were no parental refusals. So, during the 3 years of programme implementation (2005–2008) only three refusals were faced among 140 selected peer educators, leaving 137 peer educators who participated in the programme (Al-Iryani *et al.* 2011).

The peer educators were trained in a 10-day (8 h/day) workshop. The training was based on the Jordanian life skills and peer education package, and peer educators were trained in providing 10 messages on HIV transmission, prevention, and common misconceptions, and 5 life skills messages. HIV messages involved the following: the first message was on the definition of HIV and causative agent; the second on whether one can recognize an infected HIV person by the way he/she looks or whether a blood test is needed, and what the window period means; the third message was on why HIV is a dangerous infection; the fourth message was on the epidemiology of HIV in Yemen, the Middle East, and in the world; the fifth was on the mode of transmission; the sixth was on modes of prevention and adopting the ABCD (abstinence, *be* faithful, use condoms, and do not use drugs) approach; the seventh was on major misconceptions; the eighth was on risk perception and whether young people can be at risk; the ninth was on how one should deal with PLWH, which stresses the importance of the right of PLWH to live free of stigma and discrimination; and the tenth message was on the role of young people in educating the community. The life skills messages were on communication and negotiation skills, self-awareness and self-esteem, decision making, respecting differences in opinions, and assertive behaviour (Al-Iryani *et al.* 2011)

#### Training of school management teams

To ensure support for peer educators at the school level, school management teams, which consisted of school principals and vice-principals, were trained during a 5-day (7 h/day) training workshop on peer education methodologies, life skills, and HIV prevention. School coordinators planned the peer activities at their schools with peer educators and management teams (Al-Iryani *et al.* 2011).

#### Implementation at school level

Before implementation, a pre-field 2-day training was conducted, where peer educators rehearsed the actual messages to be conducted at the school level. The 10 HIV messages and 2 life skills messages (self-esteem and assertive behaviour) were the major topics covered by peer educators. Peer educators conducted educational sessions as an extra-curricular activity once a week for 90 min in a class room setting. Peer education sessions were planned in a way that each student would receive two (90 min) sessions in addition to brief sessions during morning school broadcast (Al-Iryani *et al.* 2011).

The peer educators used 70 × 50 cm posters, where each message was displayed on one poster. Also, peer education sessions were conducted during youth summer activities in the same classroom style. After the end of the second session, participants received leaflets on the 10 messages, as well as hats and T-shirts with a message: ‘Protect yourself with abstinence and knowledge’. The slogan was chosen by the peer educators; the knowledge part referred to the knowledge on condoms and knowledge on avoiding drugs (Al-Iryani *et al.* 2011).

#### Magnitude of intervention

The number of schools receiving the programme was increased gradually, starting in 2005 with the training of 27 peer educators reaching 500 students in 5 schools. Then in 2006 a team of 50 peer educators were trained from 20 schools reaching 3800 students. In 2007, the existing team of peer educators reached 4000 students in the same 20 schools, and in 2008, a team of 60 peer educators was established and 1000 students from 27 schools were targeted (Al-Iryani *et al.* 2011).

## METHODS

### Theoretical model

The theoretical model used in designing, implementing, and analysing the process evaluation included the theory of Diffusion of Innovations (Rogers 2003). Diffusion of Innovations considers that an innovation can be new information, an attitude, a belief, or a practice or any other object that is perceived as new by the individual or the community and can be diffused to a specific group (Steckler & Linnan 2002). An innovation is communicated through certain channels over time among members of a social system (here, the school). A central point in this theory is the use of opinion leaders as ‘change agents’ (Oldenburg & Glanz 2008).

### Reaching a consensus on process evaluation questions

The choice of the process evaluation instruments and the questions to be asked in each instrument was done in a participatory manner in several stages. The first stage was during brainstorming sessions between the principal researcher and the school coordinators in Aden. The second stage was during a 4-day training of data collectors. During this training, the data collectors were trained in conducting focus group discussions (FGDs) and in conducting in-depth interviews (IDIs) with young people. The training workshop for qualitative data collectors provided them with the research skill they needed, and also resulted in refining the questions to be asked.

### Sample and instruments

Multiple instruments were used to collect process data, while ensuring that all 27 schools were represented by the participants. Participants included 54 male and female peer educators, 32 male and female students who participated in the programme, 4 school principals, 4 school social workers, and 4 parents. Instruments included 12 FGD and 12 IDI.

The objectives of the 8 FGDs with the 54 peer educators were to evaluate the programme from their perspective, study the effect of life skills on their life and the life of their peers, and come up with recommendations to improve the intervention. The objectives of the 4 FGDs with the 32 students was to evaluate the methodology of peer education from the perspective of the targeted students in relation to: frequency, timing/length/place of sessions, participatory/interactive approach, and space of classroom; and whether an enabling environment existed: space, number of students per sessions, place (in schools, in summer camps). In the focus groups with students recommendations to improve upcoming programmes were also asked. The objectives of the 4 IDIs with social workers and the 4 IDIs with school principals were to explore their overall opinions on the programme, to know their role in the programme implementation, and to come up with suggestions for improvements, scaling up, and sustainability of the programme. In regard to the 4 IDIs with parents, the objective was to assess their acceptance of the peer education intervention, and to learn their perception of the effects of peer education on their children and how they estimated the impact of the peer education on the students' relationship with his/her family and on their other social relations (Table 1).

**Table 1.** Process evaluation instruments of school-based intervention

Target group	Objective/questions	Tools
Targeted students (32 male and female students)	*Objectives* (1) To evaluate the methodology of peer education in relation to frequency, timing/length/place of sessions, participatory/interactive approach, and space of classroom; if enabling environment existed: space, number of students per sessions, and place (in schools and in summer camps) (2) To come up with recommendations to improve upcoming programmes *Questions* (1) What do you think of HIV education programme in general? (2) How do you view the positive and negative aspects of the programme? (3) To what extent the sessions were participatory enough? Did you have a chance to raise sensitive questions? (4) To what extent the sessions were interactive? How could they be improved? (5) Was the environment enabling (place, number per classroom, light, etc.)? (6) Do you think some of your friends are in need of the programme and why? (7) If outside school, who do you prefer to give you HIV and life skills messages? (8) What are your recommendations to improve the programme?	4 FGDs (2 male and 2 female FGDs)
Peer educators (54 male and female peer educators participate in the 8 FGD)	*Objectives* (1) To evaluate the programme from their side (2) To study the effect of life skills on their life and the life of their peers (3) To come up with recommendations to improve the intervention *Questions* (1) How do you evaluate your experience with the programme? (2) To what extent do you think the programme has impact on you? (3) How the programme affected your life skills and practices with the family as well as friends inside and outside schools with examples? How has it affected your life beyond peer education? (4) To what extent did the programme affected your targeted colleagues with regard to life skills and practices? (5) To what extent did you have the chance to educate friends outside schools? (6) Do you have friends who are engaged in high-risk behaviour? If yes, where you able to approach them with what you know on life skills and HIV prevention? (7) To what extent did the subject of homosexuality was raised during your sessions? (8) To what extent did the subject of condom as a protective method was raised during your session? What was the feedback from students on this subject? (9) What are your recommendations to improve the programme?	8 FGDs (4 male and 4 female FGDs)
Key informants: School principals	*Objectives* (1) To explore their overall opinion on the programme (2) To know their role in the programme implementation (3) To come up with suggestions for improvements, scaling up, and sustainability of the programme *Questions* (1) What do you think about the programme? (2) What have been the strengths and weaknesses of the programme? (3) What was your role in the programme? (4) How do you see the role of youth in HIV prevention and life skills education? (5) How could you view the role of social workers in the development of life skills and youth practices? (6) What are your suggestions to improve the programme?	4 IDIs
School social workers	*Objectives* (1) To explore their overall opinion on the programme (2) To know their role in the programme implementation (3) To come up with suggestions for improvements, scaling up, and sustainability of the programme *Questions* (1) What do you think about the programme? (2) What is your vision to improve the programme in future? (3) What is your recommendation in the sustainability of the programme? (4) How do you see your future role as social worker?	4 IDIs
Parents of peer educators	*Objectives* (1) To assess their acceptance for the peer education intervention (2) To know the effect of peer education on their children with regard to: • relationship between the peers and their families • relationship between peers and surroundings *Questions* (1) Were you consulted on the involvement of your son/daughter? (2) In your opinion, what is the impact of peer education on your son/daughter? (3) To what extent do you think peer education has impacted your son/ daughter's relation inside and outside the family	4 IDIs

FGD, focus group discussion; IDI, in-depth interview.

### Data collection and analysis

#### Qualitative data

IDIs (12) and FGD (12, which included 86 students and peer educators) were facilitated by nine data collectors (6 females and 3 males), who were trained in a 4-day workshop. All FGDs and IDIs were conducted during February 2009. FGDs and IDIs were conducted over two consecutive sessions, with a 10 min break in-between, where refreshments were served, and casual discussions were stimulated by data collectors to further build trust with participants. All interviews and FGDs were tape-recorded and then fully transcribed. The transcripts were translated into English, and analysed line by line and coded thematically.

#### Quantitative data

Some quantitative results were used from the quasi-experimental evaluation, which included a random sample of 2510 students from the 27 high schools. These are mainly related to the following measure:

*Receiving peer education and whether students think its beneficial:* whether a student was targeted by the intervention or not was assessed by a closed question on whether the student had ever attended or heard of AIDS education by peers in his/her school; yes/no options were available. Those answering yes were moved to the next closed question on whether they considered peer education in schools beneficial, with the following options: beneficial; beneficial to some extent; not beneficial.

### Ethical considerations

Written consent for the study was obtained from the Director General of the Education Office in Aden. Verbal consent was obtained from all study participants. Issues related to consent seeking (anonymity, confidentiality, and voluntarism to participation) were all addressed prior to initiation of FGDs and IDIs. In addition, the Ethics Committee of the Faculty of Medicine and Health Science, Sana'a University, granted the ethical approval to conduct this study.

## Results

The themes emerging from the analysis of the FGDs and IDIs were grouped into the following categories:

- Students’ experiences of the intervention
- Acceptance of the programme by parents
- The intervention as viewed by school principals and school social workers
- Effect of life skills on peer educators and students
- Addressing high-risk behaviour and condoms in classroom setting
- Addressing high-risk behaviour and condoms out of classroom setting
- Difficulties faced during implementation

### Students' experiences of the intervention

The results from the quantitative study (quasi-experimental evaluation) revealed that among 78.6% (1964/2498) of the students who stated that they had received peer education in schools, 76.6% (1505/1964) considered it as beneficial and 21.7% (426/1964) considered it as beneficial to some extent. Only 1.7% (33/1964) of the students considered peer education as not beneficial (Al-Iryani *et al.* 2011).

In FGDs, there was a consensus among targeted students on the importance of the school-based peer education:

It is a very important programme because it addresses educating the society in general and youth in particular who are the future of any country. (A female student)

#### Role of peer educators as viewed by targeted students

Targeted students repeatedly mentioned the important role that peer educators played in the intervention. Most of the discussion was on how well the peer educators were trained, which affected positively the comprehension of messages:

The peer educators are well trained and have good communication methods to communicate information to us. (A female student)

Peer educators were also well received by targeted students because of their positive and pleasant attitude, and their patience in answering questions:

The peer educators are friendly, hard workers, energetic, patient, knowledgeable about AIDS, and never get tired of our questions. (A male student)

Peer educators had also a key role in stimulating participation of targeted students at classroom level:

The proof that the sessions are interactive is that those students who are usually sleepy in the routine classes become active in the education session. Even, hesitated and very quiet students become active and participate in a good way. (A male student)

### Acceptance of the programme by parents

All parents mentioned that their written consent for their children to participate in the peer education activities was asked by the programme coordinator from the Aden education office. They explained that their major concern was the effect of the programme on learning achievements. The school coordinators team has played a key role in advocating with parents to agree on their children's participation in the programme. As one mother reported:

I agreed after extensive discussion with school administration and their strict confirmation that this activity will not interfere with my son's study.

School principals had also indicated that effective communication with parents resulted in acceptance of the programme:

We gained the trust of parents and families. (A school principal)

All parents highly appreciated the positive effect of the peer education intervention on their children. However, they indicated that they would prefer future interventions to be conducted during summer vacation, so academic achievement would not be affected.

### The intervention as viewed by school principals and social workers

#### Role of school principals and social workers and their general reaction on the interventions

School principals and social workers indicated that the peer education interventions had a positive impact on peer educators, students, social workers, and teachers.

This is the best programme I had ever known although I had participated in many training courses. The programme is good in everything, starting from the idea to the trainers and the selected peer educators. (A school social worker)

Both school principals and school social workers were trained as part of the intervention on HIV prevention, youth participation, peer education, and life skills education. They did not take part in the actual delivery at the classroom level, but facilitated the implementation. School principals were the contact focal points between the school and the project coordinator, who was also part of the school coordinators committee and was a senior advisor to the director general of the education office in Aden. School social workers described their role in selecting peer educators to be trained, and selecting students to receive the peer education sessions:

Everything in the programme is good: the idea itself, its good consequences in raising the awareness not only for students but the community as well and its possession of all elements of success. (A school principal)

#### The concept of adolescent-led extra-curricular activities

The peer education methodology was also accepted by school principals and social workers:

The idea of having educators from the same age and educational level of student is great because they are more likely to be open with each other. (A school principal)

#### Life skills as a success factor

Life skills education was a unanimously mentioned factor leading to the success of the interventions. They suggested that peer education and the life skills interventions should be started as early as seventh grade. They believed that life skills education is a successful methodology to decrease risky behaviours among adolescents.

### Effect of life skills on peer educators and students

The school-based intervention had a clear effect on the life skills of peer educators, mainly, communication skills, assertive behaviour skills, decision making, self-esteem, and self-efficacy. This effect was repeatedly reported by peer educators and parents of peer educators.

#### Effect of life skills education on peer educators

Peer educators discussed passionately what impact life skills training had on them. They reported that life skills had positively influenced their attitude towards others and their role in life:

Learned to be more responsible, faithful, patient, flexible, good listener, respect other's opinion, able to negotiate with older and younger people. (A male peer educator)

Major comments of parents of peer educators' on the intervention were on how their children's life skills had changed dramatically. They mainly mentioned life skills related to communication, negotiation, accepting other opinions, and self confidence:

They became, self-confident, and decisions makers for themselves. (A peer educator's parent)

School principals also acknowledge the important effect the intervention had on building life skills of peer educators:

Trained peer educators are self confident, broad minded, good communicators not only for AIDS education but also in many life aspects. (A school principal)

#### Effect of life skills education as reported by targeted students

There was a consensus among students about the importance of the programme with regard to improving their knowledge on HIV prevention and transmission and attitudes towards PLWH; however, changes in life skills were less frequently mentioned:

The education helped me a lot to be assertive in my behaviour, to be proud with who I am, and to express myself very well. (A female student)

### Addressing high-risk behaviour and condoms in classroom setting

#### Male-to-male sex

Discussing male-to-male sex as a high-risk behaviour was a sensitive topic to discuss at classroom level, as one peer educator said:

I approach homosexuality only superficially because it is a very difficult and sensitive issue. (A male peer educator)

Female peer educators were more reluctant to discuss male-to-male sex as a risk behaviour relative to male peer educators.

#### Condom use

Male peer educators were more open to discuss the use of condoms than female peer educators. Female peer educators repeatedly reported that they were intimidated by the questions raised on condoms:

Girls made me embarrassed by their questions about condoms. (A female peer educator)

### Addressing high-risk behaviour and condoms out of the classroom setting

Peer educators reported that it was much easier for them to discuss high-risk behaviour and condom use with their peers out of the school setting. They thought that they succeeded in some cases in educating their peers to adapt less-risky behaviour, and had little effect in other cases:

Some of my friends said they are now using condom to protect themselves; and some are asking me where they can get tested for HIV. (A male peer educator)

We approached several girls with high-risk behaviours and explained the ways of HIV transmission and prevention. Some quitted such relations, some are now relying on condoms, while others continue practicing unprotected sex. (A female peer educator)

Peer educators indicated also that there were some rare nonsexual high-risk behaviours, such as sharing the glass for body carving, where girls carve the name of their boyfriends on their hands with a sharp glass. Female peer educators reported that although some of their peers stopped such practices, others continued. Overall, peer educators discussed the use of condoms with much more ease than discussing male-to-male sex, which is even a harder topic to tackle among female peer educators.

### Difficulties faced during implementation

Several constraints faced peer education in the classroom setting. Time was mentioned as a major constraint by both peer educators and targeted students. They all stated that more time should be given to the sessions as many questions remained unanswered after the session ended. Other constraints mentioned were the shortage of screens to display slides and the non-availability of printed material on HIV and life skills.

## Discussion

This process evaluation aimed to highlight the factors that facilitated or inhibited the peer education intervention in the 27 schools of Aden, and to provide a deeper understanding of the quasi-experimental study outcomes, which revealed that the school-based peer education intervention succeeded in improving knowledge on HIV transmission and prevention and in improving attitudes towards PLWH (Al-Iryani *et al.* 2011). Given the conservative cultural context in Yemen, the setting where the intervention was conducted, it was necessary to understand the factors that enabled as well as impeded implementation, and to assess acceptability among targeted students, school management, and parents; and in addition, to shed more light on the effects of the school-based intervention on the life skills of peer educators and targeted students.

The result of the process evaluation revealed that the intervention was highly accepted by parents, school management, and by students themselves. The main elements of success had been the involvement of school management and parents, and the high-quality training received by peer educators. This process evaluation has also indicated that the school-based interventions positively impacted on the life skills of peer educators, mainly by improving communication, assertive behaviour, self-esteem, decision making, and negotiation. This enabled peer educators to be ‘opinion leaders’ and ‘change agents’ among their peers. Peer educators were assumed to have this role by influencing not only their peers in schools for whom the activities were organized (active diffusion), but also others of relevance in the peer's environment (out-of-school peers) through an informal (passive) diffusion.

The high acceptance of the intervention by parents was mainly due to the key role of the school coordinators and the project coordinator in advocating with families. Interestingly, the main concern was not educating on HIV prevention, but rather the potential adverse effect on scholastic achievement as perceived by parents. This is an important finding, as the school-based peer education has been labelled by the Education Office in Aden as life skills and peer education programme rather than a sex education programme. Labelling as a ‘sex education’ programme might have doomed the failure of peer education intervention in the context of Yemeni schools. This ‘life skills’ label of the intervention was also the sentiment which came forward from interviews with school principals and social workers. The capacity building opportunities with training and advocacy workshops of school principals and school social workers had created an enabling environment for the intervention. Their high acceptance of the peer intervention has facilitated implementation at the classroom level as well as acceptance from families. School coordinators, who have acted as resource persons for hard-to-answer questions at classroom level, had contributed to the success of the implementation. This is in line with existing research, which documents the important role of adult support in school-based peer education (Strange, Forrest, Oakley, & the RIPPLE Study Team 2002; Visser 2007).

There is existing research, which has studied the implementation of HIV education in schools, mainly through process evaluation (Flisher, Ahmed, Jansen, Mathews, Klepp & Schaalma 2009; James, Reddy, Ruiter, Mccauley & van den Borne 2006; Oakley, Strange, Bonell, Allen, Stephenson, & the RIPPLE Study Team 2006; Markham, Basen-Engquist, Coyle, Addy & Parcel 2002; Strange *et al.* 2002; Visser 2005). All of these studies revealed that the fidelity of implementation at the class room level had a major impact on the outcome of the intervention.

The fact that the majority of students considered peer education sessions beneficial and had appreciated the participatory nature of the sessions reflects the important role of peer educators in the successful implementation of peer education sessions at the classroom level. It also emphasizes the fidelity of the programme. Previous research has also shown that peer-led school-based interventions were more effective when they were participative and skills-based (Oakley *et al.*, 2006). There is also previous research in Yemen, which documents the positive impact of peer-led interventions for HIV prevention (Al-Iryani, Al-Sakkaf, Basaleem, Kok & Borne 2010).

The FGDs had provided a more in-depth understanding of the quasi-experimental study (Al-Iryani *et al.* 2011), which revealed a lower level of knowledge on condom use and male-to-male sex among female students compared with male students, as female peer educators were more reluctant to discuss these issues compared with male students. This gender difference is a reflection of the traditional Yemeni setting, which gives more freedom to boys to discuss sensitive sexual issues compared with girls, and is similar to existing research among young people in Yemen (Al-Iryani *et al.* 2010; Al-Serouri *et al.* 2010).

The difficulties faced during implementation at the classroom level which are mainly related to time and provision of necessary visual display material are two issues that will have to be tackled if peer education and life skills are to be taken to scale in Yemen. Visual display materials would not be available in the majority of Yemeni schools, due to poor funding. For this reason, peer educators have to be trained on using existing posters. The time allocated for the sessions and the frequency of the sessions are real hindering factors in any school-based peer education intervention (Ebreo, Feist-Price, Siewe & Zimmerman 2002), and thus should be addressed in future interventions.

The present process evaluation has some limitations. The first limitation concerns the fact that it was conducted after the impact evaluation, so there is an inherent bias on the results of the FGDs and IDI (Oakley *et al.* 2006). The second limitation is that peer educator and targeted students were not asked direct questions on the effect of the peer education and life skills on their sexual behaviour and practices. There might be a missing link between the consequence of having such a large improvement in life skills among peer educators and how it was translated in the adoption of less-risky behaviour or in continuing non-risky behaviour (Flisher *et al.* 2009; Yankah & Aggleton 2008).

### Recommendations

It is recommended that life skills education is integrated within the Yemeni school curriculum in the long term and as an extra-curricular activity in the short term. The life skills and peer education training package could be used as a national training manual among in and out-of-schools young people. Several researchers might disagree with this recommendation as many studies have shown that there is no significant direct relation between acquiring life skills education and decreasing high-risk behaviour or a decrease in seroprevalence of HIV (Yankah & Aggleton 2008). However, we can argue that previous studies looking at this relation had several confounding factors, such as fidelity of the life skills education programmes, coverage, and dosage delivered (frequency of life skills sessions). There is existing research revealing that the development of effective school-based life skills programmes focusing on HIV and AIDS is a sound investment towards the sexual health of young people (James *et al.* 2006). Behavioural change is a long-term process, and it may not have a linear relationship with life skills education, but needs to reach a certain saturation point with life skills to induce a change in behaviour.

## Conclusion

This process evaluation, which was theoretically based on the Diffusion of Innovation Theory (Rogers 2003), has revealed that the ‘life skills’ labelling of the HIV prevention peer education intervention was key in the acceptance of the intervention in such a very conservative setting like Yemen. Participation and training of school management, communication with parents, and support from project coordinators had created an enabling environment for its implementation. Life skills training was pivotal in building the capacities of peer educators, which enabled them to conduct successful peer education sessions at the classroom level, and thus become ‘opinion’ leaders among their peers.
